# An inducible Cre mouse line to sparsely target nervous system cells, including Remak Schwann cells

**DOI:** 10.1186/s13064-020-00140-y

**Published:** 2020-02-20

**Authors:** Darshan Sapkota, Joseph D. Dougherty

**Affiliations:** 1 Department of Genetics, Washington University School of Medicine, Campus Box 8232, 4566 Scott Ave, St. Louis, MO 63110-1093 USA; 2 Department of Psychiatry, Washington University School of Medicine, St. Louis, MO USA

**Keywords:** Cre, Tamoxifen, Sparse, Remak, Schwann, Nervous system

## Abstract

Nerves of the peripheral nervous system contain two classes of Schwann cells: myelinating Schwann cells that ensheath large caliber axons and generate the myelin sheath, and Remak Schwann cells that surround smaller axons and do not myelinate. While tools exist for genetic targeting of Schwann cell precursors and myelinating Schwann cells, such reagents have been challenging to generate specifically for the Remak population, in part because many of the genes that mark this population in maturity are also robustly expressed in Schwann cell precursors. To circumvent this challenge, we utilized BAC transgenesis to generate a mouse line expressing a tamoxifen-inducible Cre under the control of a Remak-expressed gene promoter (*Egr1*). However, as *Egr1* is also an activity dependent gene expressed by some neurons, we flanked this Cre by flippase (Flpe) recognition sites, and coinjected a BAC expressing Flpe under control of a pan-neuronal *Snap25* promoter to excise the Cre transgene from these neuronal cells. Genotyping and inheritance demonstrate that the two BACs co-integrated into a single locus, facilitating maintenance of the line. Anatomical studies following a cross to a reporter line show sparse tamoxifen-dependent recombination in Remak Schwann cells within the mature sciatic nerve. However, depletion of neuronal Cre activity by Flpe is partial, with some neurons and astrocytes also showing evidence of Cre reporter activity in the central nervous system. Thus, this mouse line will be useful in mosaic loss-of-function studies, lineage tracing studies following injury, live cell imaging studies, or other experiments benefiting from sparse labeling.

## Introduction

Although glia are as abundant as neurons in the vertebrate nervous system and are essential for neuronal health and function, they are relatively less studied than neurons. In the peripheral nervous system (PNS), glial cells called Schwann cells are most commonly known for the formation of the myelin sheath, which insulates and protects axons and ensures that nerve impulses travel quickly and efficiently [1]. Some Schwann cells, however, do not form myelin, and these nonmyelinating Schwann cells are among the least studied cells in the nervous system. Remak Schwann cells (RSCs), a class of nonmyelinating Schwann cells, ensheath small, 0.5–1.5 μm diameter axons, such as C fiber nociceptors in sciatic nerves and form Remak bundles [2]. It is now accepted that RSCs provide trophic support to unmyelinated axons [1] and they are also implicated in nerve regeneration [2] and tumorigenesis [3]. RSCs and myelinating Schwann cells share a common pool of progenitors, which express many known molecular markers of mature RSCs [4]. This has precluded the generation of RSC-specific Cre lines and thus hindered progress in understanding the function of these cells.

When a single cell type-specific *cis* regulatory element is not readily available, intersectional BAC (bacterial artificial chromosome) transgenesis can be used to moderate the expression of a transgene driven by a promiscuous *cis* regulatory element [5–7]. This is achieved by using a second transgene that precludes the expression of the first one in some cells. Most often, this is done by crossing two transgenic lines together to create a genetic gate. However, this is less efficient as only a fraction of the progeny has both transgenes. We previously demonstrated that multiple BACs can be integrated into the same locus, yet still show independent transgene expression [8]. Using a combination of these two strategies, we report a Cre/Flpe bi-transgenic mouse line that can be used to sparsely target cells in the nervous system, including RSCs in the PNS and astrocytes and neurons in the central nervous system (CNS).

## Results

### Generation of *Egr1-Cre*-ER^T2^; *Snap25-Flpe* mice

**Fig. 1:**
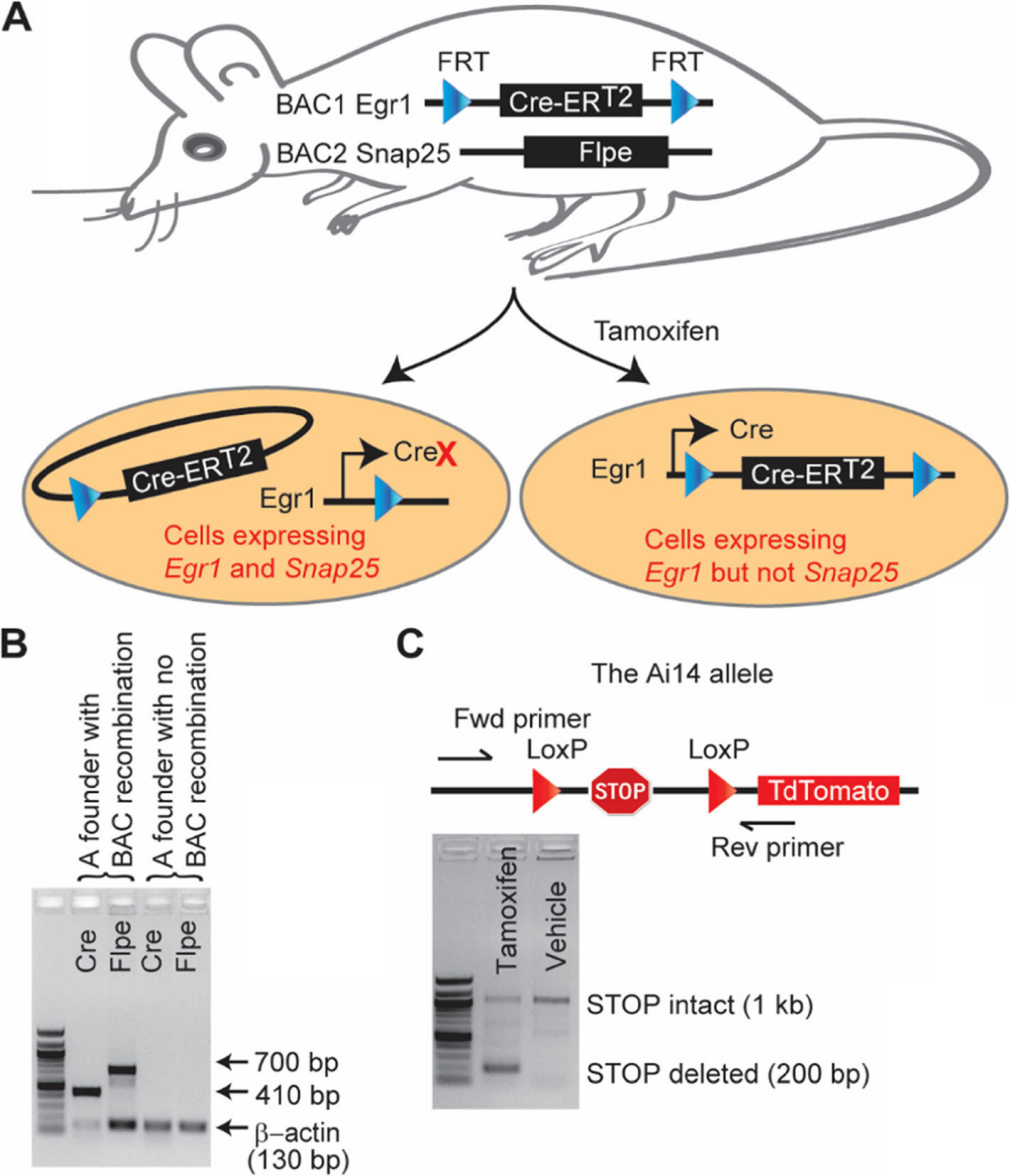
Generation of ECSF mice. **a** Intersectional transgenesis. The two BACs shown were co-injected into mouse eggs to generate double transgenic mice. In neurons and other cells with active *Snap25* promoter, the Flpe recombinase excises the FRT-flanked Cre cassette, thus inhibiting Cre expression. Remak Schwann cells and other cells with active *Egr1* promoter but inactive *Snap25* promoter express Cre upon Tamoxifen treatment. **b** PCR genotyping of founders. Representative founders with or without the Cre and Flpe transgenes are shown. **c** Cre activity in ECSF mice. ECSF mice were crossed to Ai14 mice, which have a LoxP-flanked stop cassette excisable by Cre (upper). The ECSF; Ai14 progeny were injected with tamoxifen or vehicle, and tail preparations were PCRed using primers flanking the Ai14 LoxP sites. Excision of the stop cassette is evident in mice receiving tamoxifen but not vehicle (lower). ERT2, Estrogen Receptor; FRT, flippase recognition target

To permit temporally-specific genetic manipulation, we utilized Cre-ER^T2^, a tamoxifen-inducible version of Cre recombinase, and drove its expression with a 92 kb BAC fragment covering early growth response 1 (*Egr1)*. *Egr1*, an immediate early gene, is undetectable in the embryonic nervous system, and is increasingly induced postnatally, culminating in a widespread expression in the adult brain [9, 10]. In the PNS, it is expressed by Schwann cell precursors during development, but confined to non-myelinating Schwann cells in the adulthood [11]. We reasoned that by using a temporally specific Cre, we could avoid developmental recombination. Further, in order to restrict *Egr1*-*Cre-*ER^T2^ expression to fewer cell types, we flanked the *Cre-*ER^T2^ cassette with Flippase Recognition Targets (FRTs), and simultaneously used a 61 kb BAC covering the neuron-specific synaptosomal-associated protein 25 kDa (*Snap25*) [12] to drive Flpe expression. Because *Snap25* is a pan-neuronal gene, we reasoned that the Cre-ER^T2^ cassette should be excised by Flpe in neurons during development. Thus, upon tamoxifen injection, Cre would only be expressed in *Egr1*-positive/*Snap*25-negative cells (e.g. RSCs) (Fig. 1a).

The two BACs were co-injected into the pronuclei of fertilized mouse eggs, and the resulting progeny were PCR-genotyped for Cre and Flpe to identify founders (Fig. 1b). Upon crossing the founders to wildtype C57BL/6 mice and genotyping multiple F1 mice, we found that the two transgenes were always co-inherited, suggesting that they were integrated into a single locus (data not shown). This feature avoids the need to maintain the two alleles independently and hence enhances the utility of the *Egr1-Cre*-ER^T2^; *Snap25-Flpe* (ECSF) mouse line.

We next tested if Cre is active in ECSF mice by crossing F1 mice to Ai14 reporter mice. The Ai14 mice have a LoxP-flanked stop cassette that prevents the expression of a reporter TdTomato fluorophore unless it is excised by Cre recombinase [13]. A PCR designed to detect the excision of this stop cassette indeed showed that ECSF mice express Cre recombinase at sufficient levels to mediate recombination upon tamoxifen induction (Fig. 1c).

### Identification of cells expressing Cre in ECSF mice

**Fig. 2:**
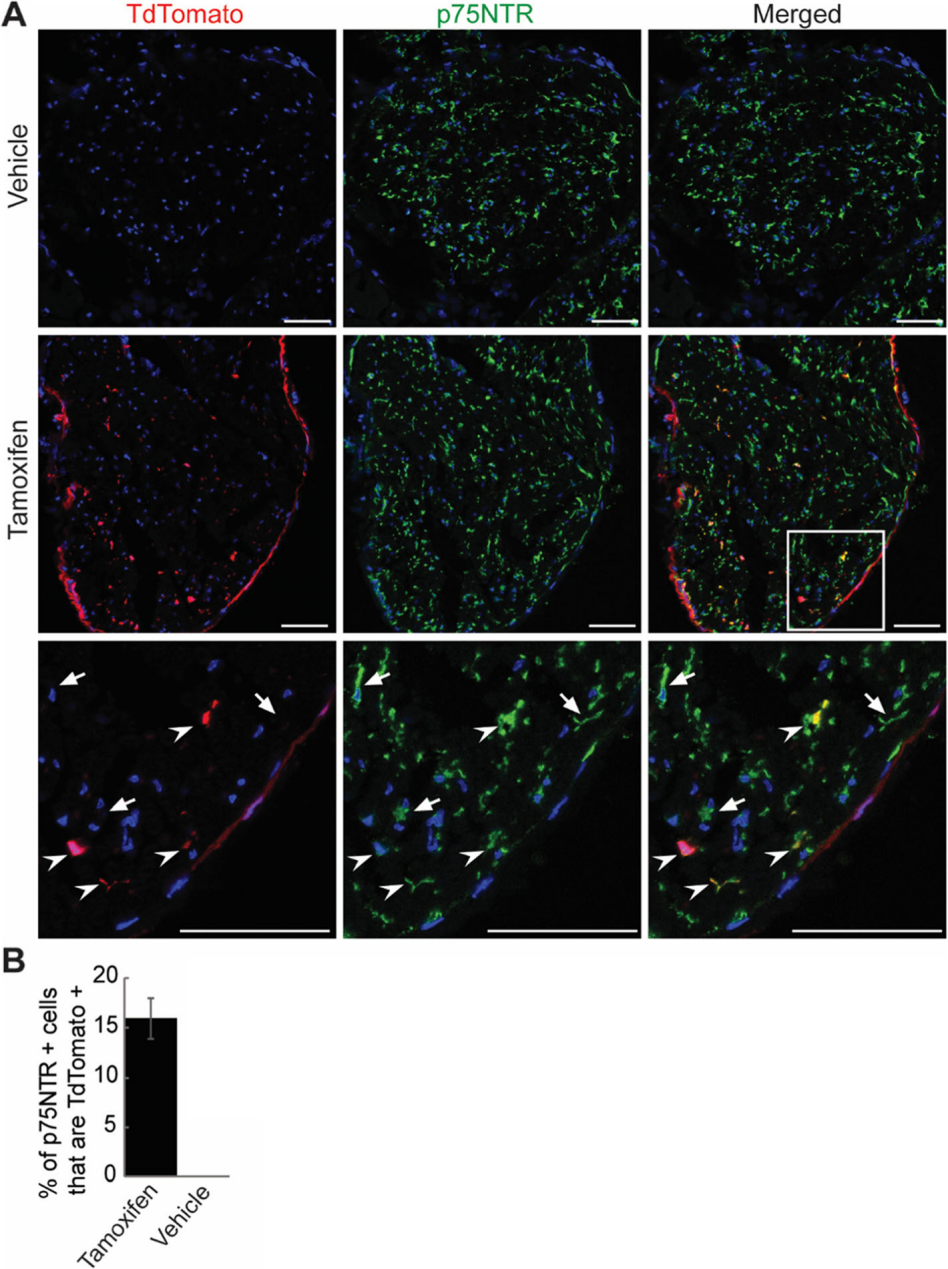
ECSF mice sparsely label Remak Schwann cells. **a** ECSF; Ai14 mice were injected with tamoxifen or vehicle and the sciatic nerves immunostained for Remak Schwann cells (p75NTR, green) and nuclei (DAPI, blue). TdTomato is undetectable in vehicle-injected mice (top) and induced sparsely in Remak Schwann cells by tamoxifen (middle). Boxed area is enlarged to depict targeted (arrowheads) and untargeted (arrows) Remak Schwann cells (bottom). Note that the perineurium surrounding the nerve is also labeled. Scales bars = 50 μm. **b** Quantification of cells in (**a**) shows 15.96% of Remak Schwann cells are targeted in tamoxifen-injected mice. Three mice; 5 sections/mouse; error bar, standard error of mean

We first asked if RSCs show Cre activity. The ECSF;Ai14 double heterozygote mice from ECSF X Ai14 cross were injected with either tamoxifen or vehicle control (sunflower oil), and their sciatic nerves were immunostained for p75 neurotrophin receptor (p75NTR), which marks Remak bundles [14]. Robust expression of TdTomato was observed in the nerves from tamoxifen-treated mice, but not in those from vehicle-treated mice, evidencing a stringent inducibility of the Cre activity (Fig. 2a). Around 16% of p75NTR-positive cells expressed TdTomato, suggesting that recombination occurred sparsely in RSCs (Fig. 2b).

**Fig. 3:**
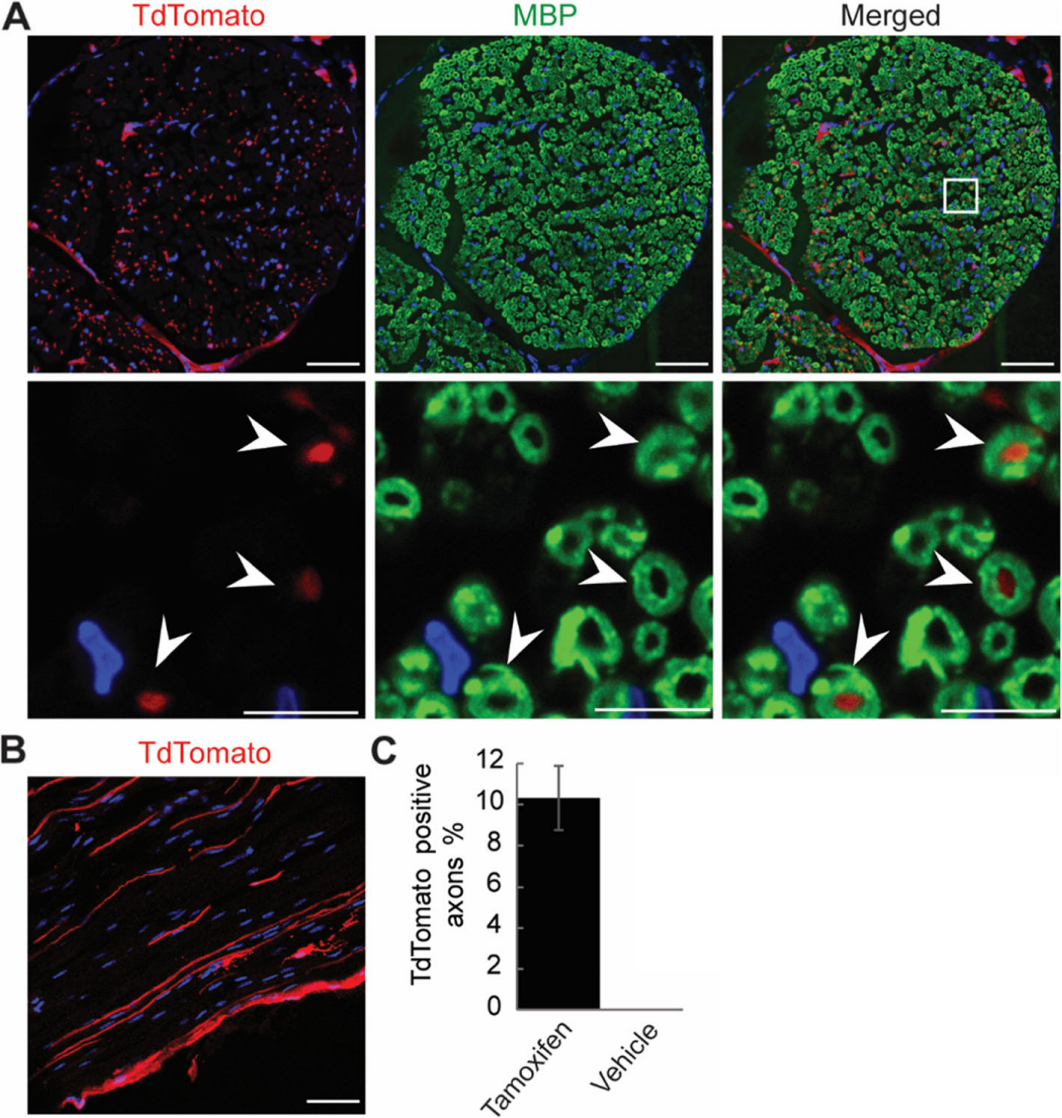
ECSF mice do not target myelinating Schwann cells. **a** Sciatic nerves of tamoxifen-injected ECSF; Ai14 mice were immunostained for myelinating Schwann cells (MBP, green) and nuclei (DAPI, blue). Lower row are enlarged views of the boxed area. A few myelin sheaths show TdTomato-expressing axons in the center (arrowheads). **b** Longitudinal view of the nerve shows TdTomato in axons. Representative images from three experiments are shown. Around 10.5% of axons were found to be targeted. Scales bars = 50 μm in upper row of a and in b; 10 μm in lower row of a. **c** Quantification of myelin sheaths with or without labelled axons shows 10.3% of axons are targeted in tamoxifen-injected mice. Three mice; 5 sections/mouse; error bar, standard error of mean

We also stained the sciatic nerves for myelin basic protein (MBP), a marker of myelinating Schwann cells [15]. While TdTomato did not overlap with MBP, it was present in the center of some of the myelin sheaths, suggesting Cre recombination in some axons (Fig. 3a). Longitudinal sections of the nerves indeed showed a few TdTomato-expressing axons (Fig. 3b). Comparison of the numbers of sheaths with and without labelled axons revealed 10.3% of the axons to be targeted after tamoxifen injection (Fig. 3c). Thus, Cre activity is excluded from myelinating Schwann cells, confirming the precision of *Egr1*-*Cre*, but present sparsely in peripheral axons, suggesting a partial insufficiency of *Snap25*-*Flpe* activity.

**Fig. 4:**
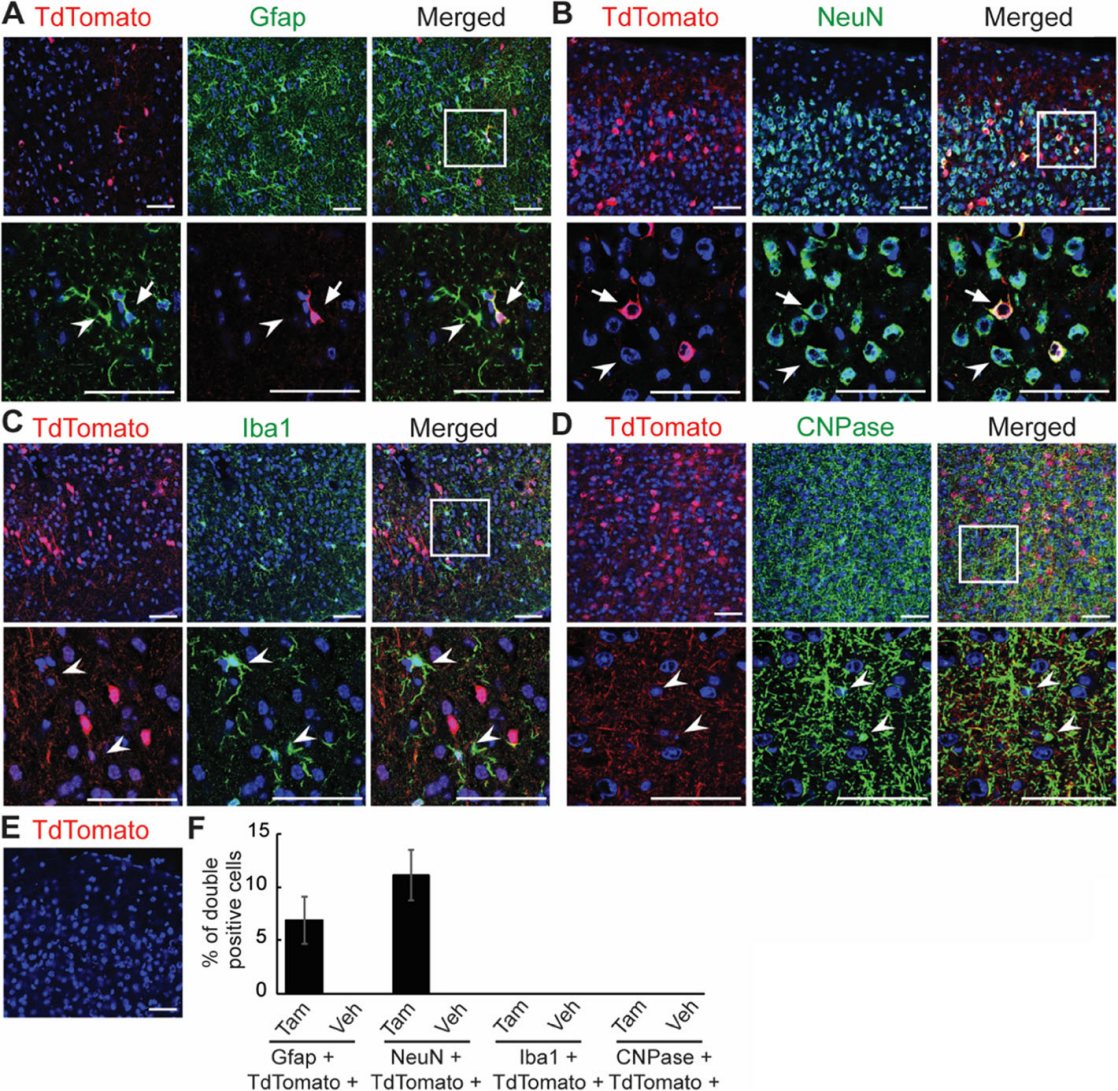
ECSF mice sparsely label astrocytes and neurons but not microglia and oligodendrocytes in the brain. **a-d** Brains from tamoxifen- and vehicle-injected ECSF; Ai14 mice were immunostained for astrocytes (Gfap), neurons (NeuN), microglia (Iba1) and oligodendrocytes (CNPase), and nuclei (DAPI, blue). Lower rows are enlarged views of the boxed regions showing representative targeted (arrow) and untargeted (arrowheads) cells in **a** and **b**, and representative untargeted cells (arrowheads) in C and D. Scales bars = 50 μm. **e** Cre activity is absent in the brain of vehicle-injected mice. Scales bar = 50 μm. **f** Quantification of cells in A-D shows that 6.8% of Gfap-positive, 11.1% of NeuN-positive, 0% of Iba1-positive and 0% of CNPase-positive cells are targeted in the cortex of tamoxifen-receiving mice. Three mice; 4 sections/mouse; error bars, standard errors of mean

Finally, we stained brain sections from tamoxifen- and vehicle-injected mice for astrocytes (GFAP), neurons (NEUN), microglia (IBA1) and oligodendrocytes (CNPase). In Tamoxifen-injected mice, TdTomato clearly overlapped with sparse astrocytes and neurons, but not with microglia or oligodendrocytes (Fig. 4a-d). Again, TdTomato was undiscernible in the brains of vehicle-injected mice (Fig. 4e). Quantification in the cortex revealed 6.8% of GFAP-positive astrocytes and 11.1% of neurons to be TdTomato positive (Fig. 4f).

## Discussion

We introduce the ECSF mouse to genetically target RSCs and other cell types in the nervous system. We show that cell targeting in this novel bi-transgenic mouse is sparse. This suggests a stochastic targeting of cells or a more uniform targeting of a specific subset of cells, for example a subset expressing *Egr1* abundantly, or both. In principle, this mouse will be useful in loss-of-function studies when only a subset of cells needs to be studied or when possible non cell-autonomous effects arising from gene deletion need to be avoided. RSCs have long been thought to transform into repair cells following nerve injuries [16]. A recent study has provided direct evidence for this by using a tamoxifen-inducible Cre mouse in lineage tracing experiments [17]. A single low-dose of tamoxifen in Plp-CreERT^2^ was found to target RSCs and a few myelinating Schwann cells [17]. We expect the ECSF mouse will also be useful in similar lineage tracing studies on RSCs. It, however, may not be ideal for studying gross pathophysiology due to a loss-of-function mutation.

Although we had expected the Cre activity to be entirely eliminated from neurons, this was not the case, possibly due to insufficient activity of the *Snap25-Flpe* BAC fragment. Yet there are occasions when a line that can reliably tag sparse neurons would be useful. For example, the Brainbow mouse has been an important reagent in studying the connectome in the brain [18]. With sparse labeling of neurons in the CNS and axons in the PNS, the ECSF mouse should be useful for similar purposes. Therefore, we have donated this mouse to the Jackson Laboratory (Stock # 033895).

## Materials and methods

### BAC recombination and pronuclear injection

A FRT-ER^T2^-Cre-ER^T2^-FRT cassette and an *Egr1* A box were cloned into a shuttle vector, which was then used to modify a *Egr1* BAC (clone ID: RP23-108C3) using homologous recombination as described previously [19]. Similarly, a Flpe and a *Snap25* A box were cloned into a shuttle vector, which was used to modify a Snap25 BAC (clone ID: RP23-290A18). BAC modifications were confirmed using restriction enzyme digestions followed by pulsed field gel electrophoresis. For pronuclear injections, a 92 kb Egr1-FRT-ER^T2^-Cre-ER^T2^-FRT BAC fragment generated from PmeI and PacI double digestion and a 61 kb Snap25-Flpe BAC fragment generated from SbfI and PmeI double digestion was purified using pulsed field gel electrophoresis followed by gel extraction (Qiagen #20021). The pronuclei of fertilized one-cell eggs of C57BL/6j mice were co-injected with a mixture of equimolar amounts of the two fragments by the Department of Pathology Knockout, Transgenic, and Microinjection Core, and then implanted into pseudopregnant foster females. The founders were genotyped using PCR. Transmission was detected in a single founder which was then crossed to C57BL/6j mice.

### Animal care

All procedures involving mice conformed to the Washington University Institutional Animal Care and Use Committee. All experimental protocols were approved by the Animal Studies Committee of Washington University.

### Genotyping

Screening of ECSF founders and genotyping of their progeny were done with PCR using Cre Fwd (CCGGTCGATGCAACGAGTGATGAGGTTC), Cre Rev. (GCCAGATTACGTATATCCTGGCAGCG), Flpe Fwd (CACTGATATTGTAAGTAGTTTGC) and Flpe Rev. (CTAGTGCGAAGTAGTGATCAGG). The Cre and Flpe alleles were further confirmed with Sanger sequencing. Ai14 mice were PCR-genotyped as described by the Jackson Laboratory for stock number 007914. Cre recombination activity in ECSF; Ai14 mice was confirmed with PCR using the Fwd primer GCGGGCCCTAAGAAGTTCC and Rev. primer TCGCCCTTGCTCACCATG to detect excision of Stop cassette.

### Tamoxifen injection

A 10 mg/ml solution of 4-hydroxy-tamoxifen was prepared in a mixture of autoclaved sunflower oil (9 part) and ethanol (1 part) with end-to-end rotation for 1 h at room temperature and stored at 4 °C in a light-proof container. Six-week old mice were injected intraperitoneally with 2 mg (200 uL) tamoxifen for 3 subsequent days. Control mice received equal volume of the oil-ethanol vehicle. Mice were processed for immunostaining after 2 weeks of injections.

### Immunofluorescence staining

Mice were euthanized and perfused with 15 ml ice-cold phosphate buffer saline (PBS), followed by 20 ml 4% ice-cold paraformaldehyde in PBS. Nerves and brains were harvested; fixed in 4% ice-cold paraformaldehyde overnight; cryoprotected with 10, 20, and 30% ice-cold sucrose in PBS for 4 h, 4 h, and overnight, respectively; and frozen in OCT (Sakura Inc). Ten Micrometre nerve sections and 40 μm brain sections were made for staining. Sections were blocked with 5% normal donkey serum plus 0.3% Triton® X-100 in PBS at room temperature for 1 h, incubated with primary antibody in block at 4 °C overnight. Antibodies and dilutions were: rabbit anti-p75NTR (Cell Signaling Technology, 8238, 1:2000), rat anti-MBP (Biorad, MCA409S, 1:100), goat anti-Gfap (Abcam, ab53554, 1:1000), mouse anti-NeuN (Millipore, mab377, 1:200), goat anti-Iba1 (Abcam, ab5076, 1:200), and mouse anti-CNPase (Millipore, mab326, 1:200). Following incubation with primary antibody, sections were washed three times with PBS, incubated with Alexa fluorophore-conjugated secondary antibodies (1:500, Invitrogen) in block at room temperature for 1 h, washed two times with PBS, incubated with 300 nM DAPI (Sigma) at room temperature for 10 m, washed two times with PBS, and mounted for confocal imaging (Perkin Elmer). TdTomato was strong endogenously and did not require an anti-RFP antibody.

### Counting of cells and axons

Cells were counted manually in tissue sections from three each of tamoxifen- and vehicle-treated mice. At least 4 sections per mouse per tissue were used, and at least 20 cells were counted per section. For axons, MBP-labelled myelin sheaths with or without TdTomato expression in the center were counted. At least 5 sections per mouse were used, and at least 30 sheaths were counted per section.

## Data Availability

The article includes all the data used to support the conclusions. The ECSF mouse is donated to The Jackson Laboratory (Stock # 033895).

## Abbreviations

BAC: Bacterial artificial chromosome
CNPase: 2′,3′-cyclic nucleotide-3′-phosphodiesterase
CNS: Central nervous system
ECSF: Egr1-Cre-ERT2; Snap25-Flpe
Egr1: Early growth factor 1
Flpe: Flippase
FRT: Flippase recognition target
Gfap: Glial fibrillary acidic protein
Iba1: Allograft inflammatory factor 1
MBP: Myelin basic protein
NeuN: Neuronal nuclei
p75NTR: P75 neurotrophin receptor
PNS: Peripheral nervous system
RSC: Remak schwann cell
Snap25: Synaptosomal-associated protein 25 kDa
